# Rapid detection of high consequence and emerging viral pathogens in pigs

**DOI:** 10.3389/fvets.2024.1341783

**Published:** 2024-02-07

**Authors:** Alison C. Neujahr, Duan S. Loy, John Dustin Loy, Bruce W. Brodersen, Samodha C. Fernando

**Affiliations:** ^1^Department of Complex Biosystems, University of Nebraska-Lincoln, Lincoln, NE, United States; ^2^Nebraska Veterinary Diagnostic Center, University of Nebraska-Lincoln, Lincoln, NE, United States; ^3^Department of Animal Science, University of Nebraska-Lincoln, Lincoln, NE, United States; ^4^Department of Food Science, University of Nebraska-Lincoln, Lincoln, NE, United States; ^5^School of Biological Sciences, University of Nebraska-Lincoln, Lincoln, NE, United States

**Keywords:** outbreaks, Oxford Nanopore MinION™ technology, viruses, surveillance, swine

## Abstract

**Introduction:**

An increasing emergence of novel animal pathogens has been observed over the last decade. Viruses are a major contributor to the increased emergence and therefore, veterinary surveillance and testing procedures are greatly needed to rapidly and accurately detect high-consequence animal diseases such as Foot and Mouth Disease, Highly Pathogenic Avian Influenza, Classical Swine Fever, and African Swine Fever. The major detection methods for such diseases include real-time PCR assays and pathogen-specific antibodies among others. However, due to genetic drift or -shift in virus genomes, failure to detect such pathogens is a risk with devastating consequences. Additionally, the emergence of novel pathogens with no prior knowledge requires non-biased detection methods for discovery.

**Methods:**

Utilizing enrichment techniques coupled with Oxford Nanopore Technologies MinION™ sequencing platform, we developed a sample processing and analysis pipeline to identify DNA and RNA viruses and bacterial pathogens from clinical samples.

**Results and discussion:**

The sample processing and analysis pipeline developed allows the identification of both DNA and RNA viruses and bacterial pathogens simultaneously from a single tissue sample and provides results in less than 12 h. Preliminary evaluation of this method using surrogate viruses in different matrices and using clinical samples from animals with unknown disease causality, we demonstrate that this method can be used to simultaneously detect pathogens from multiple domains of life simultaneously with high confidence.

## 1 Introduction

The pork industry serves as a major source of protein representing one-third of meat consumption worldwide (1, 2). Currently, the world population is expected to double by 2050 (3) with pork production to increase from 110.5 million metric tons to 128.9 million metric tons by the year 2031 (4). As a result, producers have intensified production systems to meet consumer demand. This increased intensification has resulted in high densities of animals, rapid animal turnover rates within confinement houses, and increased genetic homogeneity among swine production facilities, potentially leading to increased disease susceptibility. As a result, an increasing rate of swine and zoonotic pathogens have been identified within the industry (5). With pork serving as a major component of the world’s animal agriculture, the consequences of infectious diseases are impactful (1). One such example was the recent outbreak of African swine fever (ASF) in China which resulted in mass euthanasia of more than 1 million animals (1). Thus, causing a huge economic shift in the world pork market (1).

To prevent outbreaks, the regulatory agencies currently conduct veterinary surveillance testing for a few high-consequence animal diseases. These diseases include the African swine fever virus (ASFV), classical swine fever virus (CSFV), pseudorabies virus (PRV), foot and mouth disease virus (FMDV), and influenza A virus (IAV-S). Surveillance programs typically rely on detection using real-time polymerase chain reaction (PCR) assays or with the use of specific antibodies coupled with enzyme-linked immunoassay (ELISA) (6). As such, prior information on genetic makeup and/or antigens is necessary for detection. However, genetic drift in pathogen genomes can prevent detection as the assays are highly specific to the genetic sequence. For example, viruses such as Influenza possess segmented genomes. Therefore, such viruses can rapidly change their antigens, virulence, and ability to replicate in host species through genetic shift (7). As a result, such viruses are able to go undetected in assays that utilize specific regions for detection. Additionally, this approach prevents the identification or detection of novel or emerging pathogens. With the emergence of novel pathogens, an alternative set of surveillance tools and methods is needed that can simultaneously monitor known and unknown pathogens.

Through the development of rapid in-field sequencing methods (8), sequencing is an attractive tool for the diagnosis and surveillance of pathogens (8). Oxford Nanopore Technology (ONT) provides new opportunities for the detection and surveillance of novel and emerging pathogens. In 2014, ONT released a platform called MinION™, a handheld sequencer with reduced sequencing cost and real-time data output (8). Nanopore sequencing differs from other sequencers as it operates by measuring ionic current as a nucleotide passes through a pore (9). Within the MinION™, the typical sequencing data yield is 10–20 Gb with a maximum sequencing time of 72 h (9). The reduction in sequencing cost, increased sequencing length, rapid turnaround, and user-friendliness of the instrument have made this technology an attractive in-field diagnostic tool for patient care and surveillance (8). In addition, ONT long-read sequencing allows for gaps within genomes from short-read sequencing to be filled (8), thus, allowing for deeper knowledge of uncompleted genomes. In this study, we investigated the potential of real-time sequencing, using the ONT MinION™ platform, to identify and monitor livestock pathogens to develop a surveillance and diagnostic tool for emerging animal pathogens using swine as a model. In this study, bovine viral diarrhea virus (BVD), bovine herpesvirus-1 (IBR), and porcine Seneca Valley virus A (SVV) were used as surrogate viruses in place of classical swine fever virus (CSFV), pseudorabies virus (PRV), foot and mouth disease virus (FMDV), respectively. Influenza A virus (IAV-S) was directly used instead of a surrogate virus. Additionally, in place of the African swine fever virus, synthetically made DNA (gBlocks) was used.

## 2 Methods

### 2.1 Growth of surrogate viruses

A monolayer of 75% confluent Nasal Turbinate Horse Adapted Serum (BT CRL-1390), on Bovine Turbinate (BT) and Swine Testis (ST) cells, were inoculated with 6 ×10^3^ TCID50/mL BVD, IBR, and SVV virus, respectively, in a 150 cm^2^ tissue culture flask. Infected cells were grown in minimum essential media (MEM; GIBCO, Grand Island, NY) at 5% CO_2_, 37°C for 1 h. During incubation, flasks were gently agitated every 15 min to ensure even distribution of the virus. After 1 h of incubation, minimum essential media was added to the flasks to bring the volume up to 30 mL. The flasks were maintained at 5% CO_2_, 37°C for 3 days, for all viruses, and observed for cytopathic effect (CPE). Once CPE reached approximately 50 to 70% of the total cells, the flasks were placed in a − 80°C freezer for 30 min. The flasks were thawed, and contents were transferred to 50 mL centrifuge tubes. The tubes were centrifuged at 2,000 rpm for 5 min and the supernatant (virus stock) was transferred to a new 50 mL centrifuge tube. In total, 1 mL aliquots of virus stock were prepared in 2 mL cryogenic tubes and stored at −80° C until used as surrogate viruses for tissue spiking. Viral copy numbers were estimated using real-time PCR Ct values, as described previously, in conjunction with the assessment of virus particle concentration using viral TCID50/mL (10–17). Information regarding the estimated virus copy number for each pure virus culture can be found in Supplementary Table S1. For African swine fever (ASF), gBlocks were synthesized from the ASF genomic sequences for selected regions (Supplementary Table S2) and were used as a surrogate for the ASF virus. The sequence information for ASF genes was sourced from the NCBI database (NCBI:txid10497).

### 2.2 Identifying surrogate viruses from different matrices and unknown samples

To evaluate the applicability of using real-time sequencing strategies to detect and identify pathogens including both viral and bacterial pathogens, viral pathogens were mixed with tissue samples at 10 mL of virus culture per 25 mL of tissue homogenate (using bovine lung tissue). Bovine lung tissue was used as matrices for BVD and IBR spiking. The resulting samples were subjected to total nucleic acid extraction as described below. Briefly, the tissue samples containing the virus particles were ground using a sample disrupter (TissueLyser II, Qiagen, Hilden, Germany) for 2 min at 18 Hz and were filtered through 0.2 μm filters (Thermo Scientific, Waltman MA, United States) to remove any host or bacterial cell contamination. Following filtration, ultracentrifugation was used to pellet virus particles at 13,000 × g for 1 h. The concentrated virus samples were resuspended in 30 L of nuclease-free water and were treated with DNAseI (Thermo Scientific, Waltman MA, United States) and RNAseA (Thermo Scientific, Waltman MA, United States) to remove any free-floating DNA and RNA before subjecting to total nucleic acid extraction using the MagMAX Pathogen RNA/DNA kit (Applied Biosystems, Waltman MA, United States), high volume extraction according to manufacturer’s protocol.

Additionally, to further validate the long-read sequencing-based method developed in this study for utility in clinical samples, this approach was evaluated using clinical samples obtained from the Veterinary Diagnostic Center at the University of Nebraska–Lincoln. The study was blinded to the lab personnel conducting the experiments for presumptive viral and bacterial pathogens in the sample. The samples had been previously analyzed and the causative agent was identified for the samples using real-time PCR analysis. Tissue samples, blinded to us, containing “unknown” pathogens of both bovine and swine origin were filtered through a 0.8 μm filter in place of the 0.2 μm filter to remove host cells and retain bacteria and viruses and were subjected to DNAse and RNAse treatment and nucleic acid extraction as described above. A change in filter size from 0.2 μm to 0.8 μm was made for “unknown” samples to recover both bacterial and viral pathogens from the same sample.

### 2.3 African swine fever gBlock experiments

Bovine tonsil samples were extracted using the protocol as described above and ASF gblock DNA fragments were pooled together at a 1:1:1 ratio and added into the extracted nucleic acid from samples at the known amount of 224 and 2,242 copies of ASF gene fragments before library preparation. Exact copy numbers for each set of ASF gBlock can be found in Supplementary Table S2.

### 2.4 cDNA synthesis for detection of RNA viruses

Extracted total nucleic acids were analyzed using high-sensitivity DNA chips and Pico prokaryotic RNA chips using an Agilent BioAnalyzer 2000 (Agilent Technologies, Santa Clara, CA, United States). The RNA present in the sample was converted to cDNA using the ProtoScript II First Strand cDNA Synthesis Kit (NEBNext Biosciences, Ipswich MA, United States) and NEBNext Ultra II Non-Directional RNA Second Strand Synthesis Module (NEBNext Biosciences, Ipswich MA, United States), according to manufacturer’s protocol with the exception of using random pentadecamers to increase viral cDNA yield as previously described by Stangegaard et al. (18). As an additional quality control step to ensure viral particles were not lost during cDNA synthesis, virus strain-specific primers for BVD, SVV, IBR, and IAV-S were used to amplify target viruses (Supplementary Table S3). The primers are shown in Supplementary Table S3 and were obtained from previous literature reportings (11, 14, 15, 17). The resulting samples containing DNA and cDNA were quantified using Denovix Fluorescence High Sensitivity Assay (Denovix Inc., Wilmington DE, United States) and were used for library preparation for sequencing on the nanopore sequencing platform.

### 2.5 Nanopore library preparation

**Figure 1:**
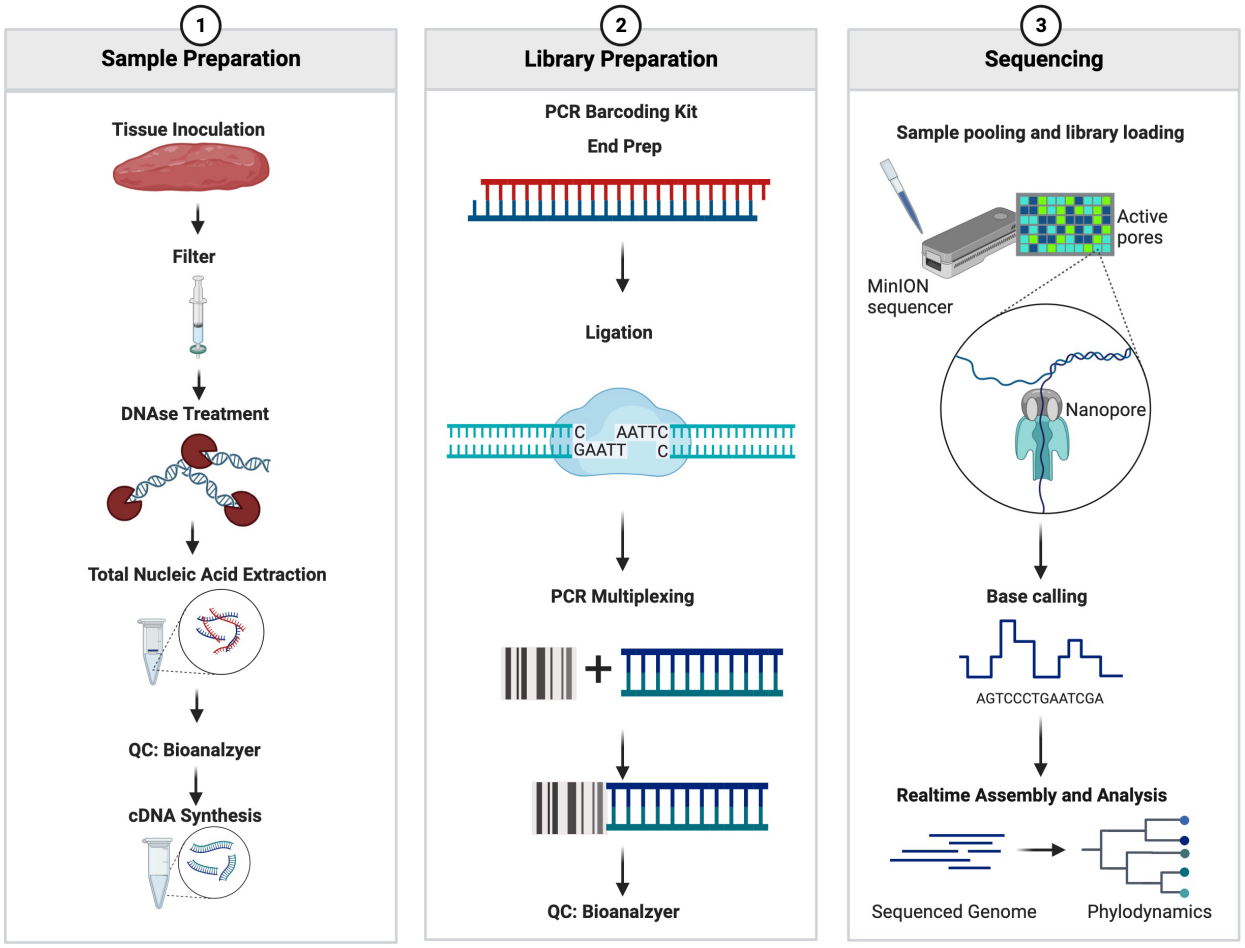
Summary workflow of the sample preparation. The figure was generated at biorender.com.

The nucleic acid mixture containing both cDNA and DNA was used for library preparation using the PCR Barcoding Kit SQK-P004 (Oxford Nanopore Technologies, Oxford, United Kingdom) with the following two modifications: ligation time was extended from 10 min to 30 min and PCR cycles were increased from 15 cycles to 18–20 cycles. Post library preparation, samples were assessed using Agilent BioAnalyzer 2000 High Sensitivity DNA Chip (Agilent Technologies, Santa Clara, CA, United States) to visualize the libraries and assess the quality and size of the libraries prepared. Library concentrations were assessed using a Denovix Fluorescence High Sensitivity Assay (Denovix Inc., Wilmington DE, United States). The library concentrations were adjusted to 0.88 nM and were pooled and sequenced on the Oxford Nanopore Technologies 9.4 or 10.3 SpotON Flow Cell (Oxford Nanopore Technologies, Oxford, United Kingdom) according to the manufacturer’s protocol. The sequencing was performed for 72 h and the read quality threshold was set to a Q-Score of >7. Fastq files were generated within the MinKNOW software and were set to generate 4,000 reads per fastq file. The full workflow can be seen in Figure 1.

### 2.6 Data analysis

Raw fastq files were barcode sorted and concatenated into a single file using the command “cat *fastq > > filename. Fastq.” The informatics tool Porechop (19) was used to remove adapters associated with each fastq file. After adaptor trimming, the fastq files were aligned to a custom database using Centrifuge (20). The custom database included all known archaea, bacteria, virus, cattle, and swine genomes present in the NCBI RefSeq database. The reference database was created using Centrifuge (20). The K-report outputs from the Centrifuge package are visualized using Pavian (21).

Data were analyzed in increments of 4,000 reads, while sequencing was performed to identify sufficient sequencing depth. Each set of fastq files generated was concatenated, trimmed, and aligned to a custom database as described above. As files were generated in sets of 4,000 reads and analyzed, a line graph was made to identify how many numbers of reads generated created a plateau, signifying that read depth had been achieved and further sequencing of each sample was not needed.

### 2.7 Availability of data and materials

Detailed information on the informatic pipeline can be found on the Fernando Lab Github page.^1^ The dataset generated and analyzed can be found under BioProject accession number PRJNA1045613 from the National Center for Biotechnology Information (NCBI) sequence read archive (SRA).

### 2.8 Identifying read depth for species identification and validation using real-time PCR

To estimate the minimum read depth required to confidently identify pathogens in a given sample, we performed an incremental analysis of reads using 4,000 read increments. Bioinformatic analysis was performed as above using incremental datasets of 4,000, 8,000, 12,000, and 16,000 reads and the taxonomic and pathogen distribution was monitored for each data set to identify read thresholds.

In addition to read-depth analysis, conventional real-time PCR analysis was performed to determine viral presence as described previously with minor modification (11, 14, 17, 22, 23). Briefly, the real-time PCR assays were slightly modified and optimized to use a commercial master mix Reliance One-Step Multiplex super mix (*Bio-Rad Laboratories*, Inc) or TaqMan™ Fast Virus 1-Step Master Mix (Thermo Fisher Scientific) as well as additional internal control system on the Biorad CFX96 Real-time PCR (*Bio-Rad Laboratories*, Inc.) or ABI 7500 FAST Real-time PCR system (Thermo Fisher Scientific). All assays were performed as part of approved protocols under the quality system of the Nebraska Veterinary Diagnostic Center that have been validated for use.

## 3 Results

### 3.1 Potential use of a novel sequence-based diagnostic and surveillance tool for viruses

**Figure 2:**
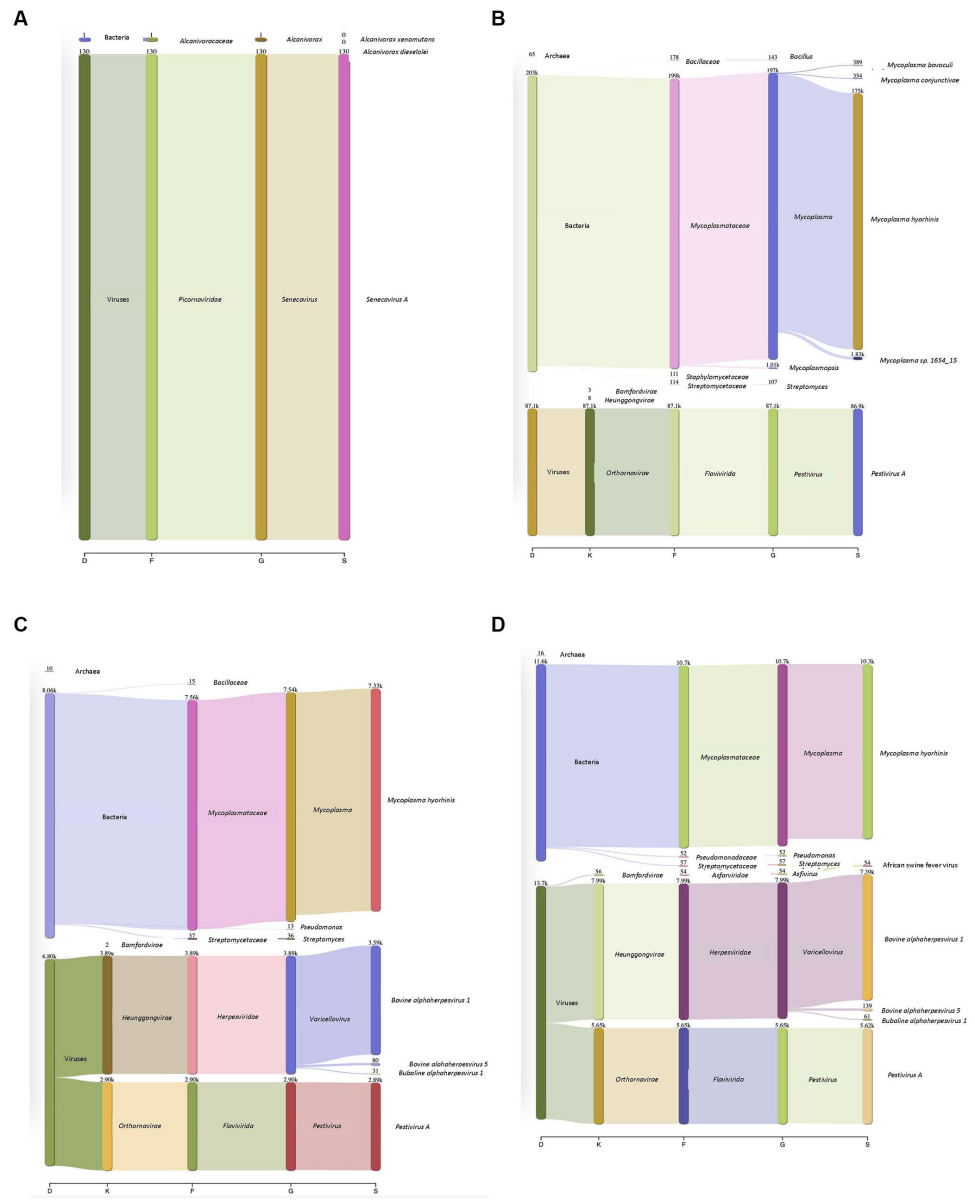
Detection of DNA and RNA viruses in different matrices. **(A)** Porcine Seneca Valley virus (SVV) identified in a spiked swine tonsil tissue sample. D–Domain, F–*family*, G–*genus*, and S–*species*. Numbers denote how many reads were annotated for each domain, family, genus, and species. **(B)** Bovine viral diarrhea virus (BVD) spiked into a bovine lung tissue sample and 86,000 reads were recovered that belonged to BVD. *Mycoplasma* was additionally identified within this sample despite filtering through a 0.2 μm filter. **(C)** Complexity of protocol was increased by increasing the number of viruses within a given sample (both DNA and RNA). Outputs showed both DNA and RNA can be detected. The viral particles were spiked onto a bovine lung tissue sample. **(D)** African swine fever (ASF) gblocks were added into a complex sample consisting of both *Bovine alphaherpesvirus* (IBR, DNA virus) and *Pestivirus A* (BVD, an RNA virus) which were spiked onto bovine lung tissue. All three types of viruses were detected.

Surrogate viruses were propagated using cell culture systems and were used to develop and validate a new protocol for the identification of known and unknown viral and bacterial pathogens from a single sample. To this end, control samples spiked with negative and positive sense ssRNA and dsDNA viruses were tested individually as well as in combination to evaluate the applicability of the method developed to simultaneously identify multiple pathogenic viruses. Individual virus detection was tested against both DNA and RNA viruses which included two surrogate viruses porcine SVV and BVD to validate that the protocol could be widely used to identify both DNA and RNA viruses within a sample. SVV (RNA virus) cell culture supernatants when inoculated onto swine tissue matrices allowed for the identification of the SVV with 19.82% of the total reads (Figure 2A), representing the target virus even with a very high level of eukaryotic DNA contamination. Additionally, a sample spiked with BVD (RNA virus) onto bovine lung tissue when tested using the approach described in this study identified 20.89% of the reads to belong to viral origin. Moreover, 51.8% of reads belonged to *Mycoplasma* (Figure 2B).

In addition to single viruses spiked onto tissue samples, combinations of DNA and RNA viruses were inoculated onto bovine lung tissues to evaluate the detection of multiple viruses within a given sample. The viral mixtures successfully identified all viral combinations inoculated. When a sample containing both a DNA (bovine Herpesvirus-1 (IBR)) and RNA (BVD) virus was analyzed through the developed sample preparation, sequencing, and analysis protocol, 19.2% of reads were identified as viral origin. Both IBR and BVD viral reads were detected in the sample at 10.96 and 8.2%, respectively (Figure 2C). Similarly, when the complexity was further increased by including African swine fever (ASF) gblocks at either 224 copies or 2,242 copies of synthetic DNA into lung tissue containing IBR and BVD, 26.6% of reads were identified as viral reads in the sample consisting of IBR:BVD:ASF_2242_ (Figure 2D). When only 224 gene copies of ASF were included within a sample matrix, only 1 read was identified as belonging to ASF. However, with 2,242 copies of ASF, 54 reads were identified as belonging to ASF, which represented 0.11% of total reads within the given sample matrix (IBR:BVD:ASF_2242_). A table of sample matrix, copy number, and read percentages can be found in Table 1.

**Table 1:** Sample matrix, copy number/ml, and read percentage identified within a given matrix.

Sample type	Surrogate virus	Spiked tissue	Estimated number of virus copies spiked (copies/ml)	Surrogate virus sequencing reads percentages	Total number of reads per virus	Percent of reads belonging to viruses out of total number of reads
Viral culture	Porcine Seneca valley virus	N/A	3.14E+08	19.82%	130	19.89%
Spiked viral culture	Bovine viral diarrhea virus	Bovine lung tissue	1.07E+11	20.89%	86,873	20.94%
Spiked mixed	Bovine viral diarrhea virus	Bovine lung tissue	1.07E+11	10.96%	2,889	19.20%
	Bovine herpesvirus 1		1.48E+06	8.20%	3,589	
Spiked mixed	Bovine viral diarrhea virus	Bovine lung tissue	1.07E+11	11.01%	7,389	26.60%
	Bovine herpesvirus 1		1.48E+06	10.58%	5,622	
	African Swine Fever gBlocks		2,247	0.11%	54	

All samples were spiked into bovine lung tissue samples. (SVV was not spiked into the tissue sample).

### 3.2 Simultaneous identification of viral and bacterial pathogens in clinical samples

To further validate our developed protocol and to evaluate the applicability of the technique developed for clinical samples, we evaluated two swine samples and one bovine sample that were “unknown” or blinded to us using the previously described protocol. In addition to samples blinded to us, we compared results found from our sequencing protocol to conventional RT-PCR. The two unknown swine samples were identified to contain porcine *Circovirus 2* (PCV2), porcine reproductive and respiratory syndrome virus (PRRSV), and porcine SVV and were consistent with results obtained through conventional RT-PCR (Supplementary Table S4). The bovine sample was identified as containing *Mycoplasma bovis*, *Mannheimia haemolytica*, *Pasteurellaceae*, and BVD pathogens and was consistent with real-time PCR results (Supplementary Table S4). As shown in Supplementary Table S4, RT-PCR was performed for unknown samples prior to ONT library prep to obtain a viral and bacterial abundance. Additionally, post library preparation, an RT-PCR was performed to identify pathogen abundance values post library preparation (Supplementary Table S4).

### 3.3 Identifying minimum read depth for accurately detecting pathogenic viruses and bacteria

**Figure 3:**
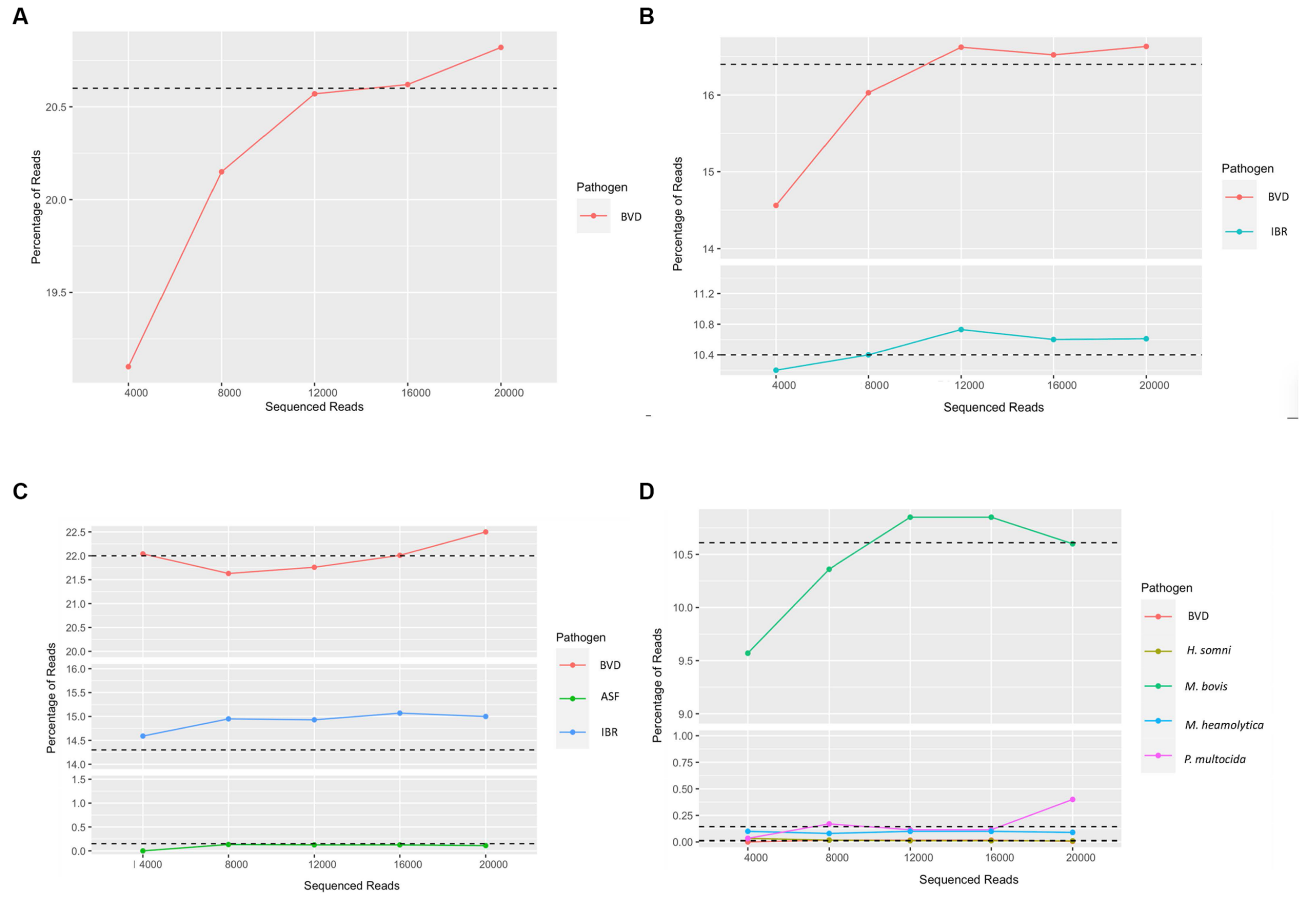
Determination of read depth required for confident evaluation of the sample. **(A)** Detection of bovine viral diarrhea virus (BVD), **(B)** Detection of bovine viral diarrhea virus (BVD) and *bovine alphaherpesvirus* (IBR), **(C)** Detection of bovine viral diarrhea virus (BVD), African swine fever (ASF), and *Bovine alphaherpesvirus* (IBR), **(D)** Unknown bovine sample used to identify potential pathogens. Outputs are the first through fifth fastq files generated. Sequencing runs were performed until all pores were exhausted to estimate sequencing depth. Sample dependency, 12,000 reads, or the first three fastq files displayed adequate depth for clinical diagnosis of the samples for a given pathogen.

To evaluate the read depth at which viral and bacterial pathogens can be accurately detected, and to reduce sequencing cost and time, we set the sequencing run to generate sequence reads in sets of 4,000 reads and analyzed data in sequential order as it came out of the sequencing run to evaluate at what read depth the pathogen detection would stabilize and provide consistent results in identifying pathogens. To this end, we evaluated sequencing runs containing different combinations of viruses and bacteria in addition to the unknown clinical samples. This approach was used on viral combinations containing both DNA and positive and negative sense ssRNA viral pathogens and bacterial pathogens. Sufficient read depth was identified when coverage of known spiked viruses reached a plateau in percentage of total reads. To this end, we assessed pathogen read abundance as a proportion of total reads as it was generated from the sequencing run in increments of 4,000 reads. This analysis identified that at 12,000 reads, we could consistently and accurately identify the pathogens present within a sample (Figure 3) depending on the starting copy number. Therefore, if sufficient read depth has been achieved, the sequencing run could be stopped and the flow cell could be washed for additional sequencing use reducing cost and saving time.

## 4 Discussion

The need for rapid identification of novel and emerging pathogens has become a requirement to ensure timely and accurate detection of pathogens to facilitate response and interventions. In veterinary virology, next-generation sequencing (NGS) has been used to test matrices for the characterization and identification of specific viral pathogens in herd screenings (6). While this is of recognition, targeting only viromes does not capture the symbiotic nature of viruses and bacteria working together to cause infection. Additionally, NGS is timely and costly especially in a diagnostic setting when result turnaround time is critical. Therefore, the use of tools such as ONT MinION provides an opportunity to mitigate these downfalls and provide a rapid diagnostic tool without any previous knowledge needed for detection. As an attempt to develop novel sequence-based tools for pathogen detection, in this study, we have developed a platform using third-generation sequencing (long read sequencings using Oxford Nanopore Technology) to identify bacterial and viral pathogens from livestock species with no prior knowledge.

### 4.1 Long-read sequencing allows for the identification of bacteria and viruses from different matrices without prior knowledge

Real-time PCR and ELISA-based diagnostic methods are widely used for rapid and accurate diagnosis of bacterial and viral pathogens, or immune responses to them. However, for such technologies, prior genetic and molecular information is needed and fails to detect novel emerging pathogens. Additionally, even for pathogens with known genetic information, recombination and mutation events can lead to current assays not being sensitive to allow for accurate detection. A good example of this is the SARs-CoV-2 outbreak that demonstrated the emergence of genetic mutations, enabling invasion of detection (24). However, utilizing whole genome sequencing approaches helps overcome such problems. Kubacki et al. (6) demonstrated the use of Next Generation Sequencing (NGS) for the investigation of viromes within clinical swine samples without prior knowledge, to identify all viruses that may be present within a given sample. This study also reported the use of NGS to help provide a deeper understanding of the viruses including the determination of genetic variants. However, Kubacki et al. (6) reported that although NGS provides many benefits such as the lack of need for prior information, accuracy, and variant analysis, the method is time-consuming and costly. Here we have developed an NGS-based platform using Oxford Nanopore Technologies to identify both viral and bacterial pathogens, which could be conducted within a 12-h time period from sample collection to diagnosis. The time from sample collection to analysis of 12 h was calculated based on 2 h for filtration, DNase treatment, and nucleic acid extraction, 30 min for quality control using Agilent BioAnalyzer 2000 (RNA and DNA), 4.5 h for ds cDNA synthesis, 3 h for library preparation, 30 min for quality control using Agilent BioAnalyzer and Denovix Fluorescence High Sensitivity Assay DNA only, and 1 h for sequencing. Analysis can be done simultaneously with sequencing ongoing. The developed protocol was evaluated using surrogate viruses spiked onto different diagnostically relevant tissue matrices.

The developed protocol is robust and can be used with multiple different tissue matrices and utilizes three different approaches to enrich for viral and bacterial pathogens targets. The approach developed allows the reduction of host cell contamination using 0.2 μm or 0.8 μm filters, which allows enrichment of virus particles (0.2 μm) or bacteria and virus particles (0.8 μm). Additionally, the concentration of filtered samples using ultracentrifugation increases detection by concentrating the samples. Finally, the DNAse I and RNase A treatment allows the removal of free-floating DNA and RNA from lysed cells. As such, these approaches help reduce host contamination and increase the sensitivity, reproducibility, and robustness of pathogen detection.

### 4.2 Validation and applicability of the method for diagnostics and surveillance

As reported by Kubacki et al. (6), the challenges of sequence-based methods compared to conventional real-time PCR-based methods for diagnostics include the time necessary for sample processing and analysis leading to delayed diagnosis and intervention. Here we have reduced the time to detection from 3 days, as reported by Kubacki et al. (6), to 12 h. In addition to rapid turnaround times, our approach has broader application with the ability to simultaneously detect both bacterial and viral pathogens. Additionally, the bioinformatically driven custom data analysis pipeline allows for data to be analyzed in real time.

Utilizing unknown samples from animals with clinical signs and samples with confirmed pathogen detection, we attempted to validate the applicability of the protocol developed for pathogen identification. To this end, we tested the protocol developed against both DNA and RNA viruses and bacterial pathogens from both swine and bovine tissue samples. Pathogens were successfully identified in these samples, potentially validating the applicability of this approach for diagnostic use. With no prior information on the pathogen needed, this approach allows laboratories to have a more robust identification strategy to identify known pathogens as well as unknown pathogens within the same sample. Even though it was not intended within this study, we were able to identify other kingdoms within the samples analyzed. Such information could provide valuable information in identifying indicator species and identifying pathogens in poly-microbial diseases rapidly. Unexpectedly, we observed a high abundance of *Mycoplasma* in the filtered samples. This observation was likely due to cell lines used to propagate viruses were discovered to have been contaminated with *Mycoplasma.* We were unable to remove *Mycoplasma* due to the fact that they are small and lack a cell wall structure (25), and as a result, we postulate, were able to squeeze through the filter used to remove host cellular contamination. Yet, even in the presence of *Mycoplasma* contamination, we were able to sufficiently identify the surrogate viruses that were used to inoculate the tissues suggesting the robustness of the protocol developed.

### 4.3 Sequencing depth and cost effectiveness

In addition to the sample processing methods developed, a real-time data analysis pipeline was developed to provide rapid analysis results for pathogen detection. To identify the minimum depth of sequence reads and the need for accurate detection to reduce sequencing time and cost, we performed analysis on the data as it is being released from the sequencing platform. To this end, we analyzed datasets generated at 4,000 read increments for viral and bacterial species present within the sample for surrogate viruses that were inoculated. The sequential analysis of reads as recovered through sequencing clearly demonstrated that a read depth of approximately 12,000 reads will provide a robust estimation of pathogens present within the sample. As such, this approach will reduce the time used for sequencing (30 min) and resources used for sequencing, thereby reducing diagnostic costs. Additionally, at this stage, the run can be stopped and the flow cell can be reused for additional sequencing at a later time. As such, the barcoding approach described above increases sample throughput and helps reduce cost per sample making the method developed broadly applicable to clinical samples and surveillance of current and emerging pathogens.

ONT MinION platform has advantages such as rapid turnaround time, portability, and capability of detecting organisms across multiple kingdoms. Additionally, multiplexing helps reduce the time and cost of sample analysis. However, the cost for a single sample processing might be higher than real-time PCR. As such if used for surveillance studies the cost may be prohibitive.

Recently ONT developed a tool, called ReadUntil, for selective sequencing, where if a read does not match target organisms of interest, the read can be ejected from the pore. Here, “unwanted” reads would be kicked out of the pore and a new read would begin sequencing once the pore has recovered (9, 26). According to Kovaka et al. (9), this can be accomplished by reversing the polarity of the voltage across the pore to eject the DNA molecule and allow for a new sequencing read to begin sooner. Furthermore, if unwanted reads are identified quickly enough, enrichment for target reads can be done solely on a computational technique (9). In a study conducted by Ong et al. (27), ONT ReadUntil (adaptive) sequencing, standard ONT sequencing, and Illumina shotgun sequencing were compared to identify which method was the most efficient in profiling the bovine vaginal microbiome while reducing host reads. Within this study, higher numbers of annotated genes were identified among the ONT ReadUntil group (adaptive sequencing), which demonstrated the advantages of ONT adaptive sequencing in samples that have a high host-to-microbe DNA ratio (27).

In addition to ReadUntil, another group has optimized the program within ONT and created an additional program called UNCALLED which also further optimizes reducing the sequencing of unwanted reads of larger fragments while continuing to refine itself during the sequencing run (9). Within this program, investigators can upload sequencing of reads they either want to enrich by allowing for the continuation of sequencing within a given pore or deplete sequencing of (unwanted reads) by the pore rejecting the unwanted read(s) (9). Here, Kovak et al.’s (9) algorithm converts stretches of signals into k-mers and uses higher probability k-mers as a query for the Ferragina-Manzini (FM) index to search against target databases (9, 26).

This approach may be helpful in reducing costs and focusing on multiple target organisms from the same sample. In addition, if host cell contamination is high, the depletion of host reads could allow for further enrichment of microbial reads. Using tools such as ReadUntil or UNCALLED will not enable the identification of unknown emerging pathogens. Thus, the approach described within our study can be applied to the detection of known and unknown pathogens and organisms in a given environment with the flexibility to amend the sequencing protocol based on user needs.

## 5 Limitations

While this protocol shows great promise in its applicability for clinical diagnosis, sample type and quality of the nucleic acids extracted could affect performance. As ONT MinION™ is developed for long-read sequencing that helps with better annotations, and extraction of short nucleic acid fragments may hinder the performance of this approach. Additionally, for more complex samples and samples with a lot of host contamination, the DNase treatment is critical and may need to be performed for a longer time or repeated. For more complex samples and samples with greater host contamination, read depth needs to be increased. One additional concern is read quality on the ONT MinION™ platform. However, recent advancements in technology have increased base calling accuracy from 98.3% to ≥ 99% (28). While ONT MinION™ allows for in-field sequencing, the protocol developed in this study needs a laboratory setting and is not feasible in the field in its current stage.

## 6 Conclusion

Here we developed a novel protocol using long-read sequencing to identify known and unknown bacterial and viral pathogens of livestock that is rapid, robust, and can be broadly applied. Additionally, we have demonstrated the protocol is robust at using known pathogens and clinical samples with uncharacterized pathogens from different tissue types and show how potential applications for this technology to be utilized for diagnosis and surveillance for both endemic and emerging diseases of livestock.

## Data availability statement

The original contributions presented in the study are publicly available. This data can be found at: https://www.ncbi.nlm.nih.gov/bioproject/; PRJNA1045613.

## Ethics statement

The animal study was approved by University of Nebraska Animal Use and Care Committee. The study was conducted in accordance with the local legislation and institutional requirements.

## Author contributions

AN: Data curation, Formal analysis, Investigation, Methodology, Writing – original draft, Writing – review & editing. DL: Data curation, Formal analysis, Investigation, Methodology, Resources, Writing – review & editing. JD: Conceptualization, Data curation, Funding acquisition, Methodology, Project administration, Resources, Writing – review & editing. BB: Funding acquisition, Supervision, Writing – review & editing. SF: Conceptualization, Data curation, Funding acquisition, Methodology, Project administration, Resources, Supervision, Writing – review & editing.

## Footnotes

^1^ https://github.com/FernandoLab/MinION_high_consequence_viral_pathogens
