# Supplementary material for: Rapid detection of high consequence and emerging viral pathogens in pigs
Source: Front Vet Sci. 2024 Feb 7;11:1341783. doi: 10.3389/fvets.2024.1341783 (PMC10879307; doi:10.3389/fvets.2024.1341783)
Supplement: Supplementary file 1 [file Data_Sheet_1.PDF]

Supplementary Table 1. Viral copy number (copies/ml) associated with each pure viral isolate.

**NAHLN Pathogen**	**Surrogate Virus**	**Genome**	**Family**	**PCR CT value after extracted**	**Viral TCID50/mL to use for extraction**	**Estimated number of virus copies spiked (copies/ml)**
Classical swine fever virus	Bovine viral diarrhea virus	RNA	*Flaviviridae (Pestivirus)*	17.98	39905.25	1.07E+11
Pseudorabies virus	Bovine herpesvirus 1	DNA	*Herpesviridae*	14.46	632455.53	1.48E+06
Porcine influenza A	Porcine influenza A	RNA	*Orthomyxoviridae*	32.56	35.00	1.20E+02
Foot and mouth disease virus	Porcine senecavirus A	RNA	*Picornaviridae*	15.00	12000.00	3.14E+08
African swine fever virus	Synthetic sequences “gBlocks”	DNA	*Asfarviridae*			
