# Supplementary material for: Rapid detection of high consequence and emerging viral pathogens in pigs
Source: Front Vet Sci. 2024 Feb 7;11:1341783. doi: 10.3389/fvets.2024.1341783 (PMC10879307; doi:10.3389/fvets.2024.1341783)
Supplement: Supplementary file 2 [file Data_Sheet_2.PDF]

Supplementary Table 2. African swine fever (ASF) synthesized gBlocks dsDNA fragment used in place of ASF virus.

Gene ID	Sequence	Copy Number
41901830	atgttgtg gcgcaatgaa attacagaat ttatggatca<br>actttccaag tattgccaag aaatcttaaa aacgtttaaa<br>cagttgcgtc ctagtgaata taaacagtac aatgaatttt<br>taacacaagt tacaccgttg ctgcaaaaaa cccctgaaaa<br>aattccagag gaggttgacc atattttcga ttacctagat<br>aacgttgaaa aaatttgtga gctactggtg catgctagct<br>caattattat tagttcaaaa atacgagaac aagtaaaaca<br>cggaatgagc ttcagctata aaaccgacct cgactccttg<br>gcgggcatcc tctctcaaaa acagtacgtg cttattcatc<br>tttcaaaaaa tattgcggcc cactatttta atacgtgttt<br>aaatcaaggg aaatccaggt tagatctcaa ggctgcctct<br>gtgttttata atagccgtcc ccggaccgca agctcagcag<br>aactatatag aaaaatgcta tacgcctatg gctcgctaca<br>agaaattaat tattatacgg aaaaagcccg aaataagacg<br>ctggatgtgg aggagagtga cagcataacg accatcgaac<br>ggacggcccg tcacaacctt tcccttatgc acccgctaga<br>agccatggga cttacctttg gggcaaccaa cacgaacgca<br>gacccggagg atctgaagga taaaacggtg ataaatttaa<br>cgcttccgca ggcaacagaa agcgtcacct accatcttaa<br>ttccctaatg cagctaaaaa aagtaagtac gacttcagga<br>ctaaatacaa acattttgaa agcgtttgat aatattattt<br>ccgcccctgt gaaaaaaaat aaaatggcct ccaagttggc<br>gcccggaatg gatgtcgtgt tcactagcga taacggaaaa<br>acattttta cgaaaaacgt tttaagcaaa aacatgctag<br>cggggcccaa agagcgggtg tttgcatata ataatctcat<br>tagtaattta aataactcct gcttcataca aaatcacaac<br>gattttttga gacagcagga ctcttggccc ttctatgacg<br>cacacaattt taccaacaaa tttttaatgc agcctatttt<br>ttcagggcag acccgtcctc ggctccaggg agccatggag<br>gccgcgcacg tagaaacgcg tcttacggca tttttgcaaa<br>gtattcaacc ttccaggcca caagatccct ctattttggc<br>ttcccccaag ttatctgctc taatcttgaa ctaa	7.13 X10^9
41901848	ttaa tcttttatca gatacctaaa accttttata<br>agtgagtcta tgagatggat catctcttga tggtcatcgt<br>aagaagcaag ctttctagca aaaacgacag cgttaaagaa<br>tttattgcgc tcgtgtctgg ataatacttt taatagcgaa<br>ccaaaacagt atttaaaaat ttggcaacag tttttttggg<br>ctgcaataaa caaacacttg atcagtgcct gcttcacttt<br>ctgatcggac atatttgccg cataacaggc ttttttaaac<br>ttagtaatat aattatgttt cgcaagtacc attaacaagg<br>aagctatggg aagctgcttt tcttggtgaa attcacgtaa<br>acatttgatg gccagtgctt ggaagacagt gtgacttatt<br>aagtcagaaa tgatagtttt catggttgta aaaatataca<br>taggattttc ttgttctgta tatagtttga aaagcttatc	6.05 X10^9

	attgcgtgaa atgatggcca tttttaatac aagatagtat<br>agtttatctt tagataaaaa tgctttgcaa gccgtgatta<br>tgtcgatgtt gttgttatga atagcgatag aaagtaatgt<br>ttctattctg aatgttttta aatgccttaa cagaggaatg<br>cagtttatgt tattatattt aacaatactg taatacaccg<br>aatcaatgac ggtcatctga gcatcaagcc gatttattag<br>caaatttaac gtttttttgg aggcatgacc tttaatggcg<br>gcactaagag cgcatagtat agtaaaattg ttaaatatat<br>tttggttaaa gagaagcagt aatattttcc ttcggttata<br>gtacgcagca tctgtgatga tgattggacg ataaacgtta<br>aaatgggtta acagcttttt aaaaaaacgg aagtagtttt<br>tttgtatcgc tgtttgcatc atcgaaataa tgagatggtc<br>agggtacgta atgggtaggt cacatgctac ctctaacaaa<br>gaataatcac ccaatctaaa ggctgtgtta aaaagcgtac<br>tatcatcata cgtatcgaac actcctgctg ttgcaaacca<br>agcaatgaga tgaatgtgcc gttccttgca agctatcgca<br>aatagggcat ttcctatgga atgtcgaaca atgtactccc<br>tatttttttc caaaatgttt tgaaaattgt atagcgttgc<br>cgcatacagt agacactcca ttctggcgtg ataattttta<br>cttttgcata tgaatagatg gaagaactcg aataattctt<br>gagaacttgt taaatgcata atgtggtgat agcttggtgt<br>agttaaatga tgtgagaaaa tgcattctat tacatctttt<br>cggttatgtt ttagtgcctg agctaaggca tattcaggct<br>caacccatag tactagtgtt tctagaattc tgatatttgc<br>ctgctttgcc aatgcatact ttaaaacact ctggttggaa<br>aacattttgt tatgaagatg gacgacagtg tccattttta<br>tgatgggacc attccagtat agtcctaaat gctgtagcag<br>atcatttgtt aggtctgagg cgtcctcggg agtcatataa<br>atatgttgca acgctttttt ctgtaaggag aacat	
41901859	atgcc atctacttta caagcacttg ctaaaaaagt<br>actggccaca ccgcatatat ctaaaaaata ttgtcagtct<br>aaggagtact gtcatatatt aaaatgttgt ggtttatggt<br>ggcatgatgc tccaattaca atttatcctt gtataaataa<br>aatattgata aaaacagcaa gctttaaaca tggtttagag<br>ttgaatgtcg cattcatgaa agctgtacag gaaaataatt<br>atgatctaat aaggctgttt attgagtggg gtgcggatat<br>caactttggg ttggccactg ttaatacaga ccgtacccgg<br>gacctgtgcc gggaactagg tgcgaaggaa gctttaagtg<br>aaaaagaaat tttagaaata tttgataaaa tacagtatat<br>taaaagtagc agtaatatta ttgtatccca tgaattatta<br>tctaataacc cccttttctt aaataatgat caattgaaat<br>taagaatgtt cgatgaacta cataaactat caatcaactt<br>tgtattacat gagatatcat ttaatgaaat gctaactagg<br>tattggtata gtatggcgat actatataac ctcaccgcag<br>ccattcaata tttttatcaa tcctacaagc attttaaaga<br>ttggcggtta atatgtgggc ttgcttataa caacgtgttt<br>gatcttcatg aaatatataa caaagagaag gttgatatgg	$9.24 X10^{9}$

	atattaatca aatgatgcag ttggcctgta tgtatgatgg<br>taattataca accatttatt attgttttat gttgggagct<br>gacattaatc gggcaatgat tacctcggtt acaaaatctt<br>gtgatggtaa cttattcctt tgtatagatt tgggggctga<br>tgtatttgaa gagagtatga aaatggcaat agaagatcat<br>aatgacgtat tagaaagtat cttatcattt aaaaattatt<br>atagttcaga cgtttctctg ttatcattaa agacgacaga<br>tccagaaaaa attaatgcct tgttagaaga agaaagttac<br>aaatcaaaaa ataggttgat atataaaagt tattga	
