# Supplementary material for: Rapid detection of high consequence and emerging viral pathogens in pigs
Source: Front Vet Sci. 2024 Feb 7;11:1341783. doi: 10.3389/fvets.2024.1341783 (PMC10879307; doi:10.3389/fvets.2024.1341783)
Supplement: Supplementary file 3 [file Data_Sheet_3.PDF]

Supplementary Table 3. Surrogate virus specific primer sequence, annealing temperature, and target fragment sized used for PCR validation of the protocol in DNA isolation and library preparation steps.

		Annealing Temperature ºC	Fragment Size bp	Reference
BVD-F	GGGNAGTCGTCARTGGTTCG	55	190	Liang 2019
BVD-R	GTGCCATGTACAGCAGAGWTTTT	55	190	Liang 2019
IBR-F	TGTGGACCTAAACCTCACGGT	60	76	Thonur 2012
IBR-R	GTAGTCGAGCAGACCCGTGTC	60	76	Thonur 2012
SVV-F	AGAATTTGGAAGCCATGCTCT	60	75	Fowler 2017
SVV-R	GAGCCAACATAGARACAGATTGC	60	75	Fowler 2017
SIV-F	AGATGAGTCTTCTAACCGAGGTCG	60	100	Richt 2004
SIV-R	TGCAAAAACATCTTCAAGTCTCTG	60	100	Richt 2004
