# Supplementary material for: Rapid detection of high consequence and emerging viral pathogens in pigs
Source: Front Vet Sci. 2024 Feb 7;11:1341783. doi: 10.3389/fvets.2024.1341783 (PMC10879307; doi:10.3389/fvets.2024.1341783)
Supplement: Supplementary file 4 [file Data_Sheet_4.PDF]

Supplementary Table 4. Unknown samples CT value prior to MinION™ library preparation and post preparation.
Estimated copy number/ml based off of previously reported literature [13, 16, 29].

	PCR CT value Pre-MinION Librabry Prep	Estimated number of virus copies (copies/ml)	PCR CT value Post-MinION Librabry Prep	Estimated number of virus copies (copies/ml)	Unknown Virus Sequencing Reads Perecentages	Total Number of Reads per virus	Percent of Reads belonging to viruses out of total number of reads
UK1 Swine	*Porcine Circovirus 2* CT=8	6.47E+11	*Porcine Circovirus 2* CT=12.72	2.26E+10	19.67%	22,293	19.70%
	Porcine reproductive syndrome virus CT=25	1.00E+05	Porcine reproductive syndrome virus CT = NA	NA	NA	NA	NA
UK2 Swine	Porcine seneca valley virus A CT=37	8.00E+01	Porcine seneca valley virus A CT=28.34	4.10E+02	0.01%	7	0.01%
UK1 Bovine	Bovine viral diarrhea virus CT=28.26	1.16E+08	Bovine viral diarrhea virus CT=35.23	1.13E+06	0.01%	1	0.01%

* colors denotes if viruses were identified with conventional diagnostic methods (qPCR) and ONT sequencing pre or post library prep.
Green denotes samples identified in both qPCR assay and ONT sequencing pre and post library prep.
Red denotes samples that were identified in pre MinION prep but could not be identified in qpcr or sequencing post prep.
