# Supplementary figures and images for: Rapid detection of high consequence and emerging viral pathogens in pigs
Source: Front Vet Sci. 2024 Feb 7;11:1341783. doi: 10.3389/fvets.2024.1341783 (PMC10879307; doi:10.3389/fvets.2024.1341783)

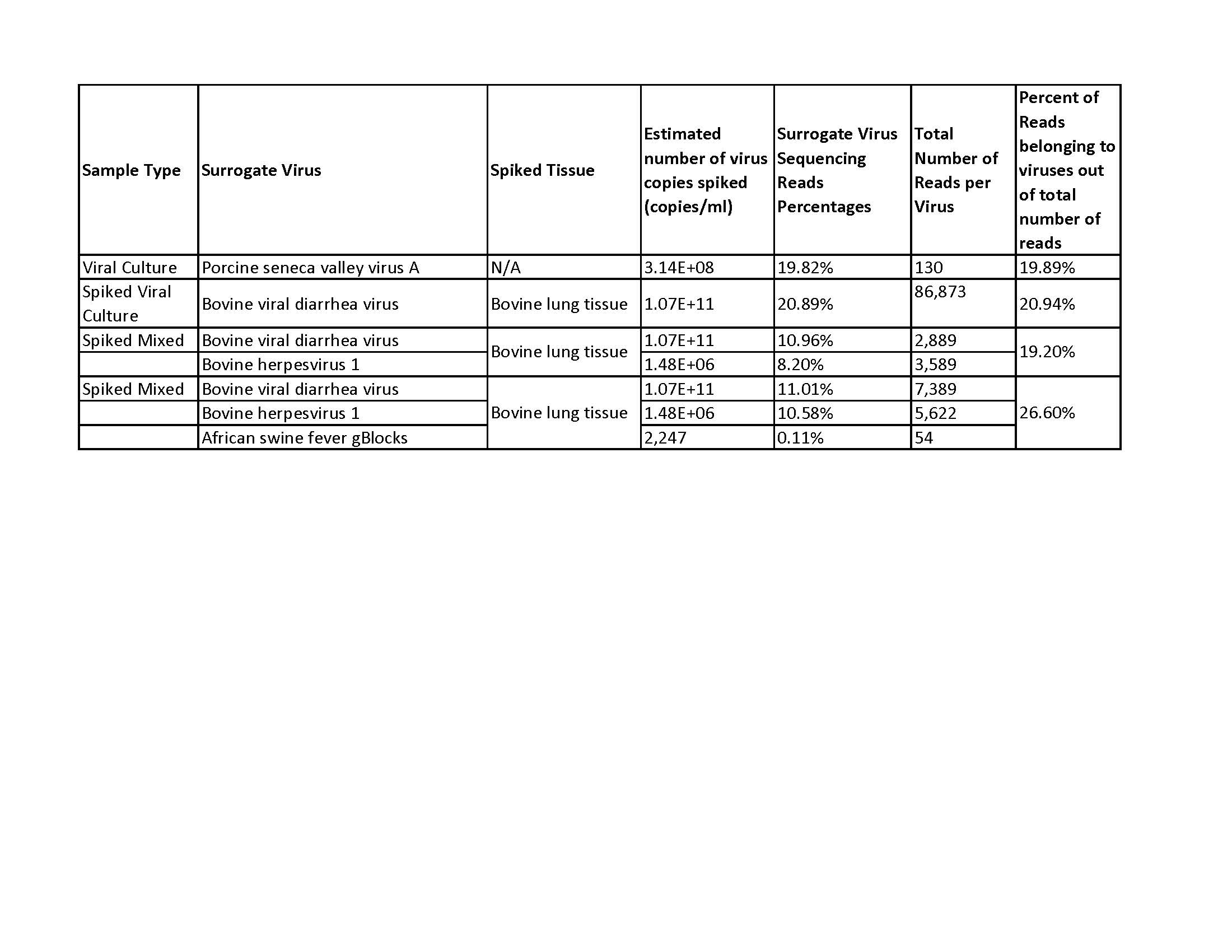

Supplement: Supplementary file 5 [file Image_1.JPEG]
